# Misdiagnosis of cerebral malaria initially as acute psychotic disorder and later as human rabies: a case report

**DOI:** 10.1186/s13104-016-2211-3

**Published:** 2016-08-11

**Authors:** Meththananda Herath Herath Mudiyanselage, Nayani Prasangika Weerasinghe, Kithsiri Pathirana, Hasini Dias

**Affiliations:** Faculty of Medicine, University of Ruhuna, Galle, Sri Lanka

**Keywords:** Cerebral malaria, Atypical presentation, Phobic spasms, Human rabies

## Abstract

**Background:**

Cerebral malaria is arguably one of the most common non-traumatic encephalopathies in the developing world. Unless the diagnosis of cerebral malaria is made promptly, the consequence could be disastrous. Even though the diagnosis of cerebral malaria can be made relatively easily in majority of cases atypical presentation can often lead to misdiagnosis or delayed diagnosis. We report a case of an uncommon presentation of *Plasmodium falciparum* infection in a 17-year-old school girl with altered sensorium, seizures and phobic spasms.

**Case presentation:**

A previously healthy 17-year-old school girl was admitted to our hospital with acute condition characterised by comatose state, recurrent seizures and phobic spasms. She initially presented to a local hospital with agitation and over talkativeness and was diagnosed as having an acute psychotic state. Few days later she became drowsy and developed recurrent seizures and marked phobic spasms which prompted the treating physician to diagnose human rabies. However, further investigations carried out in our unit (including rapid antigenic test for *P. falciparum* and peripheral blood smear) were positive for *P. falciparum*. She was treated as for cerebral malaria with intravenous quinine and discharge from hospital with no residual neurological deficit.

**Conclusion:**

Atypical presentation of cerebral malaria can often lead to misdiagnosis. This patient presented with encephalopathic illness with phobic spasms was initially misdiagnosed as human rabies. Therefore, the physicians in malarial endemic areas should be vigilant of similar presentations and should consider cerebral malaria as a possibility.

## Background

Cerebral malaria is a life threatening complication of falciparum malaria especially when managed inappropriately. It is characterised by rapid onset of unarousable coma associated with or without seizures [1]. Few decades ago, cerebral malaria was often considered as a strong possibility when a patient presented with fever with altered sensorium in Sri Lanka [2]. However, with dramatic decline of incidence of malaria during last decade, cerebral malaria is no longer considered as a strong possibility in such a clinical situation [3].

We report a case of an uncommon presentation of *Plasmodium falciparum* infection in a 17-year-old Sri Lankan school girl, who initially presented with acute psychotic state followed by altered sensorium and phobic spasms (hydrophobia and aerophobia) typically observed in human rabies [4].

## Case presentation

A previously healthy 17-year-old school girl was admitted to our hospital with acute condition characterised by comatose state and seizures. The family reported that she fainted while in the school 2 weeks earlier and was referred to the General Hospital of Hambanthota, in Southern Sri Lanka. Following unremarkable examination and baseline investigations, the patient was discharged. However, following day morning her family noticed some unusual behaviour of the patient including agitation and over talkativeness. As her agitation worsened over next few days, she underwent psychiatric consultation, in which an acute psychotic state had been diagnosed and antipsychotic was prescribed. She did not have symptoms such as fever, headache, chills or rigors at this stage of the illness. Over next 2 days she gradually became drowsy and developed several episodes of seizure. With these new symptoms she was readmitted to the same regional hospital. On admission she was reported to be drowsy with Glasgow Coma Scale (GCS) of 8/15, and had hypertonia with opisthotonus posture. She had exhibited phobic spasms including hydrophobia and aerophobia (positive fan test). Furious rabies was suspected as the most likely diagnosis on the basis of her acute encephalopathic state, phobic spasms and an unconfirmed dog bite history around a month ago. As there was no definitive treatment she was managed conservatively and the family was informed the poor prognosis. Devastated by this acute illness which has no cure; her family sought second opinion from our hospital.

On admission to our unit, she was febrile with a temperature of 39.6 °C, heart rate of 120/min, respiration of 38/min, and blood pressure of 134/82 mmHg. She was drowsy with a fluctuating level of consciousness, incoherent, and not cooperative with the examination. Pupils were equal (3 mm) and reactive to light. Fundoscopic examination revealed no abnormalities. No obvious jaundice, enlarged lymph nodes, spleen, or cranial nerve palsies, were observed. She had opisthotonus posture with hypertonia and exaggerated reflexes. She also had obvious phobic spasms including phobic spasm with air (positive fan test). Involuntary jerky movements suggestive of myoclonic jerks were observed at frequent intervals throughout the hospital stay.

**Fig. 1:**
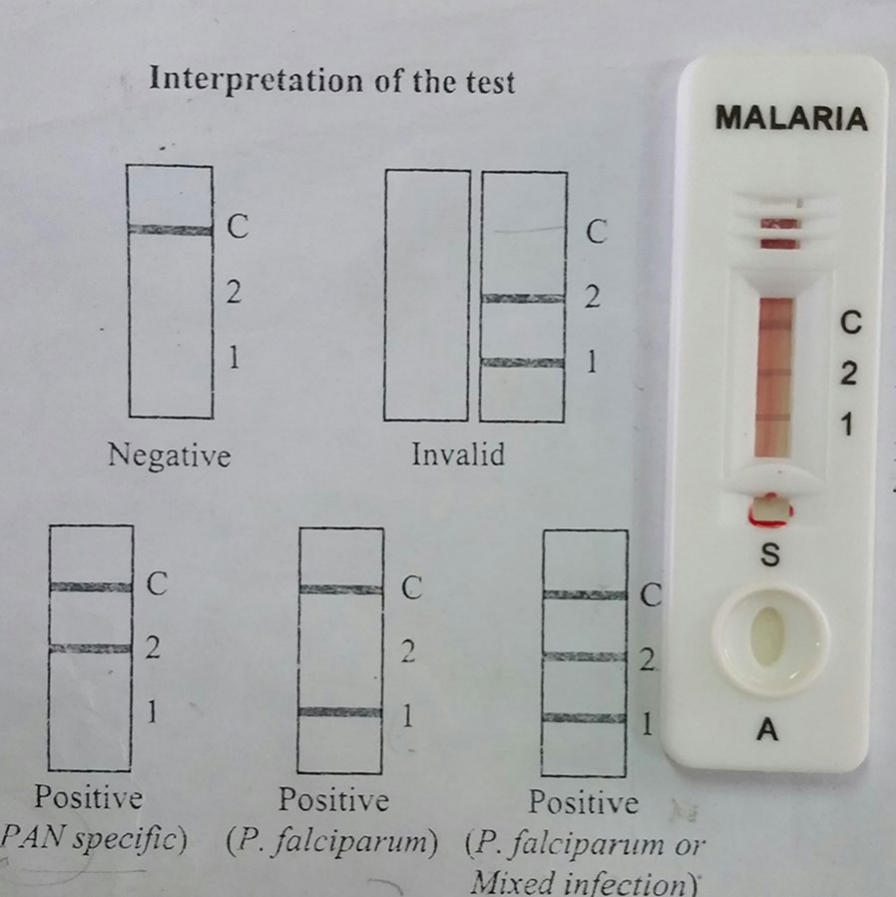
Blood film of this patient—positive for *P. falciparum* ring forms

Investigations showed white blood cell count of 7300 cells/μl with 74.3 % neutrophils and 18.3 % lymphocytes, haemoglobin concentration 13.2 g/dl, platelet count 156,000 μl^−1^, creatinine 38 μmol/l and erythrocyte sedimentation rate 32 mm/h. Urgent non contrast computed tomography showed no significant abnormality and cerebral spinal fluid examination was unremarkable with protein of 43.4 g/l and 2–4 lymphocytes/mm^3^. The electroencephalogram showed frequent generalized 4- to 6-Hz spikes in the back ground of slow-wave discharges. A rapid antigenic test for *P. falciparum* and peripheral blood smear for malarial parasite were performed as the patient was from geographical area traditionally considered as malarial endemic area. Antigen test was positive and the peripheral blood smear revealed the presence of *P. falciparum* ring forms (Fig. 1).

Soon after the diagnosis was made, the patient was started on intravenous quinine with intravenous dextrose. She also was treated with sodium valproate to control the seizure activities. She started to show signs of improvement on the following day and her confused mental state gradually improved over 3 days. On Day 07, she was discharged with a good mental status. The patient was reviewed monthly for 3 months and during the follow up visits she did not have any residual neurological deficit or cognitive impairment. Sodium valproate was stopped 3 months later as she did not have seizures anymore.

## Discussion

Malaria is one of the most important parasitic diseases of humans, and its main neurological complication, cerebral malaria is arguably one of the most common non-traumatic encephalopathies in the world [5, 6]. Cerebral malaria is primarily caused by *P falciparum* [5]. However, there are infrequent reports of cerebral malaria associated with *P. vivax* infection [7]. The mechanisms involved in the pathophysiology of the cerebral lesion are not totally clear, however, engorgement of cerebral capillaries and venules with parasitized red blood cells (RBC) and non-paratised RBCs is thought to be the most likely reason for the clinical features of cerebral malaria [1, 6, 8].

Even though malaria is common in most of the tropical countries affecting 234 million people with 2.5 million deaths per year, there has been a dramatic reduction of case load in Sri Lanka during last decade [3, 9]. Number of cases plunged from 210,039 in the year 2000 to just 23 cases in 2012 (99.9 % reductions) [3]. As malaria is virtually eradicated form Sri Lanka, it is no longer considered by most physicians as a possibility for an acute encephalopathic illness. However, there are sporadic cases reported in some part of Sri Lanka [3]. Therefore, clinicians have to be vigilant about its presence.

This case illustrates the difficulty in diagnosing atypical presentation of condition which is getting rarer and rarer. Absence of any obvious pointers favouring the diagnosis of cerebral malaria is a reason for difficulty in diagnosis. Our patient had many atypical features. First she had an acute psychotic state without any prodromal symptoms such as fever, body ache or headache. Fever is a cardinal symptom of malaria and most patients with malaria experienced fever before the onset of other symptoms related to cerebral dysfunction [5]. However, in some patients fever could be totally absent or dominated by features other than fever [10]. Furthermore, recognising cerebral malaria based on fever and its pattern may lead to delay in diagnosis as typical fever pattern with chills were seen only in 55.55 % patients [11]. Acute psychotic state observed in our patient has been reported in previous studies too [6, 11]. Like in our patient altered sensorium could be present from the outset, or might develop slowly over a period of several days [12]. Signs of irritability, restlessness or psychotic behaviour are other the initial manifestations of cerebral malaria [11, 12]. Seizures are common in children and in adults it is seen in occasionally [13].

The most unusual manifestation observed in our patient is phobic spasm which is typically described in patients with human rabies [4, 14, 15]. Presences of phobic spasm along with symptoms of encephalitis led to wrong diagnosis of human rabies in our patient. Rabies remains top in the lists of differential diagnosis of encephalitis in rabies endemic area like Sri Lanka. Phobic spasms in the form of aero- and hydrophobia are important and useful clinical feature in diagnosing human rabies [4]. Furthermore, phobic spasms are considered to be pathognomonic of human rabies and they have not been previously described in patients with cerebral malaria [16]. Possible mechanism for phobic spasm in human rabies is functional modification of limbic system due to pro-inflammatory molecules produced by infected neurones [14]. Same mechanism could occur in patients with cerebral malaria as malarial parasites can induce inflammation in neurones in the brain and brain stem similar to viral involvement in rabies. However, it is interesting to find why phobic spasms have not been previously observed or reported in patients with cerebral malaria. One possible explanation is that the phobic spasms could be a rare phenomenon of cerebral malaria or occurrence of them may depend on other factors such as concomitant use of antipsychotic medications. But it is possible that some of the patients with cerebral malaria with phobic spasms may have been wrongly diagnosed as rabies and hence not reported in literature.

## Conclusion

This case illustrates difficulty in diagnosing atypical presentation of cerebral malaria presenting as an encephalopathic illness with phobic spasms. Phobic spasms which are typically seen in human rabies were observed in our patient with cerebral malaria. This is the first time that presence of phobic spasm as a significant manifestation was described in a patient with cerebral malaria. Physicians in malarial endemic areas as well as rabies endemic areas should vigilant of patients presenting with encephalopathy and should consider cerebral malaria as a possible diagnosis in patients presenting with phobic spasms and encephalopathy.

## References

[1] Idro R, Marsh K, John CC, Newton CRJ (2010). Cerebral malaria; mechanisms of brain injury and strategies for improved neurocognitive outcome. Pediatr Res.

[2] Senanayake N, Roman GC (1992). Neurological complications of malaria. Southeast Asian J Trop Med Public Health.

[3] The National Malaria Control Programme, Sri Lanka. Guidelines on malaria chemotherapy & management of patients with malaria 2012. http://www.malariacampaign.gov.lk/precentation/treatmentguide.aspx. Accessed 28 May 2016.

[4] Hemachudha T, Ugolini G, Wacharapluesadee S, Sungkarat W, Shuangshoti S, Laothamatas J (2013). Human rabies: neuropathogenesis, diagnosis and management. Lancet Neurol.

[5] Greenwood BM, Bojang K, Whitty CJ, Targett GA (2005). Malaria. Lancet.

[6] Idro R, Jenkins NE, Newton CR (2005). Pathogenesis, clinical features, and neurological outcome of cerebral malaria. Lancet Neurol.

[7] Sarkar S, Bhattacharya P (2008). Cerebral malaria caused by *Plasmodium vivax* in adult subjects. Indian J Crit Care Med.

[8] Storm J, Craig AG (2014). Pathogenesis of cerebral malaria—inflammation and cytoadherence. Front Cell Infect Microbiol.

[9] World Health Organization. Factsheet on the world malaria report 2013. http://www.who.int/malaria/media/world_malaria_report_2013/en/. Accessed 28 May 2016.

[10] Deb T, Mohanty RK, Ravi K, Bhagat BM (1992). Atypical presentations of falciparum malaria. J Assoc Physicians India.

[11] Mishra SK, Mohanty S, Satpathy SK, Mohapatra DN (2007). Cerebral malaria in adults—a description of 526 cases admitted to Ispat General Hospital in Rourkela, India. Ann Trop Med Parasitol.

[12] Newton CR, Warrell DA (1998). Neurological manifestations of falciparum malaria. Ann Neurol.

[13] Mishra SK, Newton CRJC (2009). Diagnosis and management of the neurological complications of falciparum malaria. Nat Rev Neurol.

[14] Hemachudha T, Laothamatas J, Rupprecht CE (2002). Human rabies: a disease of complex neuropathogenetic mechanisms and diagnostic challenges. Lancet Neurol.

[15] Wilson JM, Hettiarachchi J, Wijesuriya LM (1975). Presenting features and diagnosis of rabies. Lancet.

[16] Kietdumrongwong P, Hemachudha T (2005). Pneumomediastinum as initial presentation of paralytic rabies: a case report. BMC Infect Dis.

